# From a Facial Hemangioma to a Critical Diagnosis: Uncovering Coarctation of the Aorta in an Infant

**DOI:** 10.1016/j.acepjo.2025.100176

**Published:** 2025-05-22

**Authors:** Kyle Conley, Inna Kaminecki

**Affiliations:** 1 Department of Emergency Medicine, Texas Tech University Health Sciences Center, Lubbock, Texas, USA; 2 Department of Pediatric Emergency Department, UMC Children’s Hospital, Lubbock, Texas, USA

**Keywords:** PHACE, hemangioma, coarctation

## 1 Patient Presentation

A 5-week-old female was brought to the emergency department by her mother with concerns about a progressively worsening facial rash over the past 4 weeks. The rash became increasingly erythematous and raised, although the surface area remained unchanged. Recently, the patient was noted to have intermittent difficulty opening her left eye. Otherwise, she was developing normally. The mother also reported pectus excavatum at birth, which was no longer noticeable. The baby was born at term, and no complications were noted during pregnancy or delivery.

**Figure 1:**
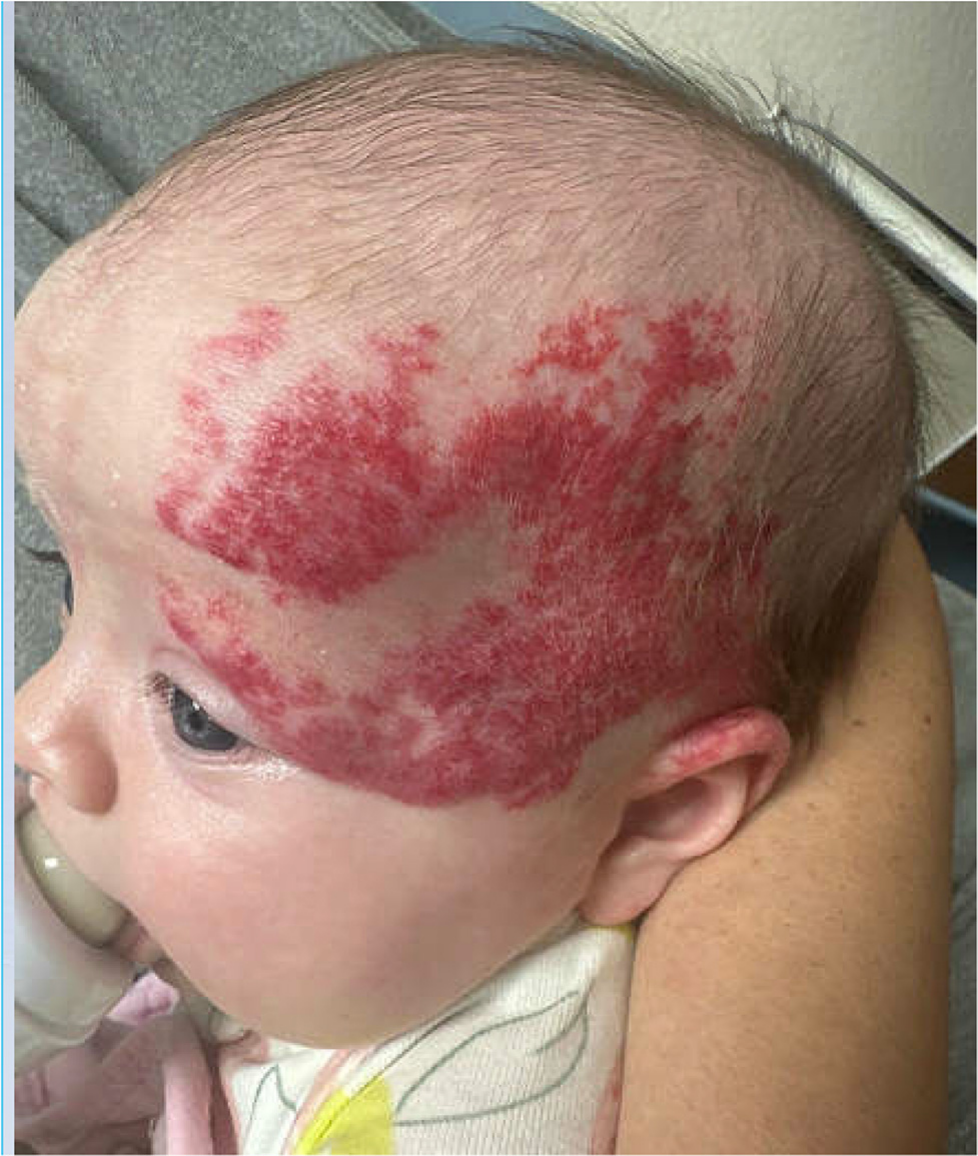
Segmental hemangioma distributed over the left forehead, temporal area, lateral upper eyelid, and posterior left ear.

On physical examination, the patient appeared well. Her temperature was 99.6°F, heart rate was 132 beats per minute, respiratory rate was 42 breaths per minute, blood pressure was 89/49 mm Hg, and oxygen saturation was 100% on room air. She had a well-demarcated, red-to-violaceous telangiectatic, dense plaque segmentally distributed over the left forehead, temporal area, lateral upper eyelid, and posterior left ear (Fig 1). Left-sided ptosis was also observed. Breath sounds were clear bilaterally, and no cardiac murmurs were noted. Neurologic examination was normal. The abdomen was soft, with the liver edge palpated 4 cm below the costal margin. The patient had mildly diminished femoral pulses bilaterally.

**Figure 2:**
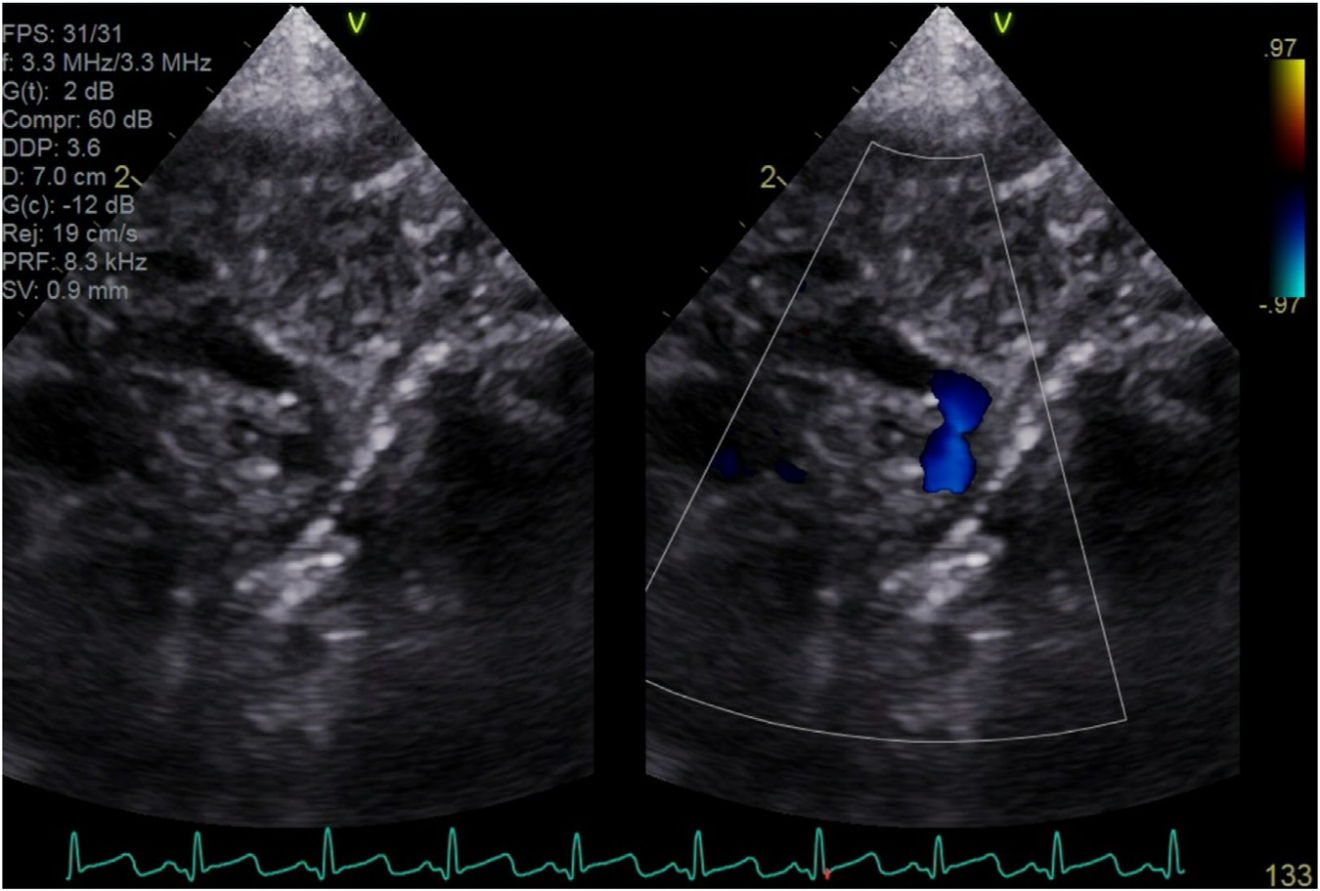
Transthoracic echocardiogram identifying juxtaductal coarctation of the aorta.

Given the distribution of the hemangioma and diminished femoral pulses, PHACE syndrome (posterior fossa abnormalities, hemangioma, arterial cerebrovascular anomalies, cardiac defects, and eye anomalies) was in the differential diagnosis. Electrocardiography revealed signs of left ventricular hypertrophy, whereas the chest radiograph was normal. Transthoracic echocardiography identified coarctation of the aorta (juxtaductal), with the descending aorta measuring 2 mm at its narrowest point (Fig 2). Additionally, the patient was noted to have a small muscular ventricular septal defect and mild concentric left ventricular hypertrophy. A cranial ultrasound showed no acute abnormalities, notably a normal-appearing posterior fossa. Dermatology was consulted. Their evaluation was consistent with facial infantile hemangioma, and topical timolol was recommended. Ophthalmology was also consulted, and a vascular malformation of the iris, hypopigmented fundus, possibly mildly anomalous left optic disc, and elevated intraocular pressure of 24 mm Hg were found.

**Figure 3:**
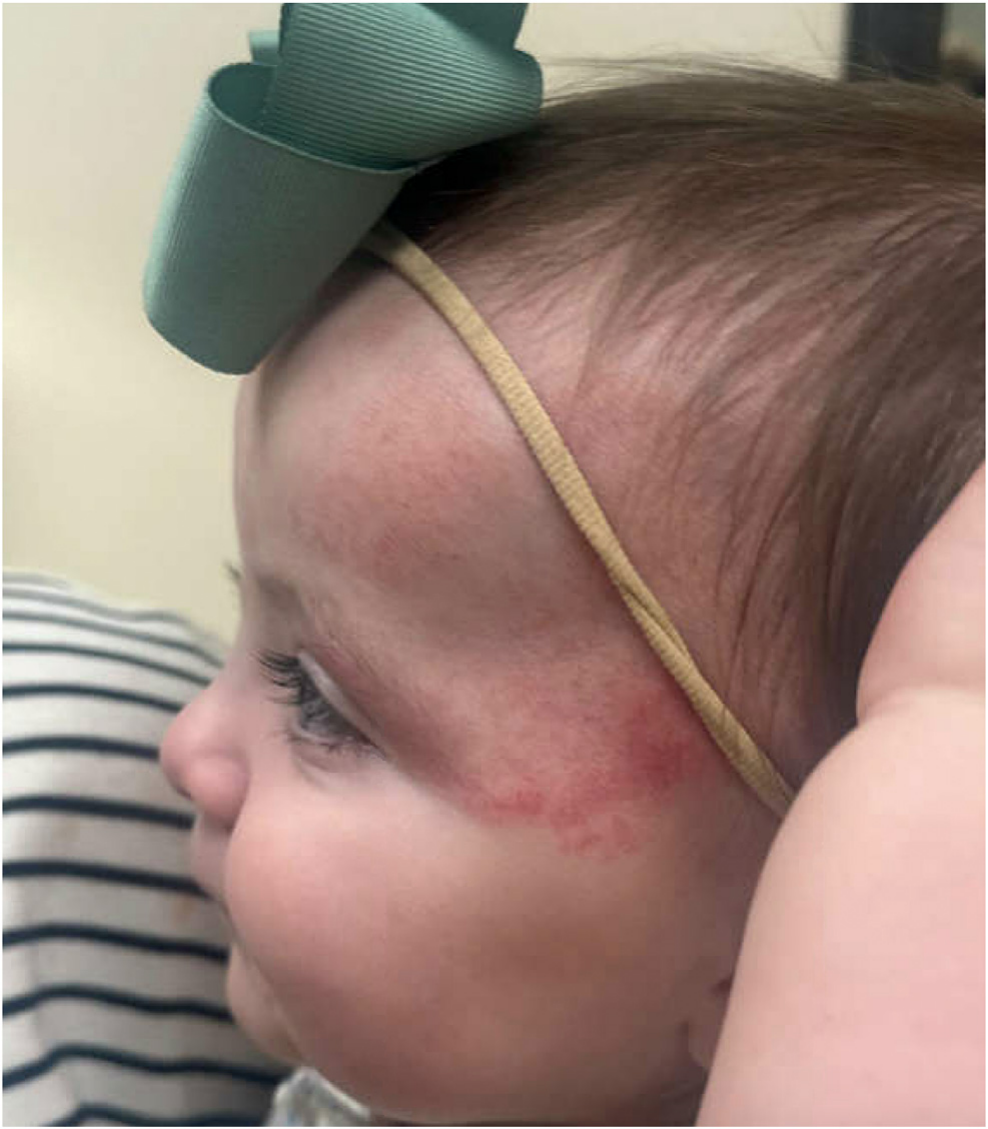
Resolving segmental hemangioma.

The patient was admitted to the pediatric intensive care unit and started on prostaglandin E1 infusion. She was promptly transferred to an outside facility for evaluation by pediatric cardiothoracic surgery and subsequently underwent surgical correction of the coarctation. Further work up at the outside hospital revealed additional findings, including internal carotid stenosis/hypoplasia, anomalous cranial and retinal arteries, hepatic hemangioma, and pectus excavatum. Advanced imaging confirmed normal posterior fossa anatomy. The patient continues to follow up with multiple specialists and has been noted to be doing well, with an improving hemangioma on oral propranolol (Fig 3).

## 2 Diagnosis: PHACE Syndrome

PHACE syndrome is a rare, idiopathic neurocutaneous disorder characterized by posterior fossa malformations, segmental infantile hemangiomas (IH), arterial anomalies, cardiac defects (commonly coarctation of the aorta), and eye anomalies.^1^ Additional ventral developmental defects, such as sternal cleft and/or supraumbilical raphe, may also occur. A multicenter, prospective study on facial hemangioma morphology and risk of PHACE syndrome found that among patients with large facial hemangiomas (>22 cm^2^), one-third were diagnosed with PHACE syndrome.^2^

Diagnostic criteria, which include major and minor criteria, emphasize the importance of an IH diameter on the head (including the scalp) >5 cm as a key feature.^3^^,^^4^ Airway assessment is also essential, as there is an association with airway hemangiomas.^5^ Facial IH is the most common, but rare cases involving the scalp, cervical, occipital, upper thoracic, trunk, and proximal upper limb regions have been described. There is an increased likelihood of PHACE syndrome with frontotemporal and frontonasal segmental IH.^6^ As in this case, the IH may initially appear as a faint precursor lesion before proliferating over time.^7^

The most common extracutaneous anomalies are head and neck vascular malformations, which are present in 70% to 90% of cases and are almost always ipsilateral to the IH.^8^, ^9^, ^10^ Due to these malformations, patients are at an increased risk of ischemic stroke.^11^^,^^12^ Headaches are common, and many patients experience hearing loss.^13^^,^^14^ A significant number of individuals with PHACE syndrome suffer from long-term developmental delays and neurocognitive impairments.^15^^,^^16^ Cardiac and/or aortic anomalies affect approximately two-thirds of patients. Coarctation of the aorta and/or an aberrant subclavian artery are most common, each affecting about 20% of patients, and they frequently co-occur. In one registry, 90% of patients with cardiovascular abnormalities also had cervical or cerebral arterial involvement.^17^^,^^18^ Eye abnormalities associated with PHACE syndrome include optic nerve hypoplasia, microphthalmia, persistent fetal vasculature, and optic disc anomalies.^4^ As in this case, IH can lead to ptosis. Additionally, amblyopia and proptosis are common findings.^19^ Endocrinopathies have also been reported, most commonly thyroid dysfunction and hypopituitarism.^20^^,^^21^

Over 300 cases of PHACE syndrome have been described, but the true incidence remains unknown.^7^^,^^22^ The condition predominantly affects females, with an approximate female-to-male ratio of 9:1.^7^ Once the syndrome is suspected, recommended screening includes an echocardiogram (with cardiac magnetic resonance imaging (MRI) if abnormalities are identified), MRI of the brain with and without contrast, magnetic resonance angiography of the head, neck, and aortic arch, as well as hearing screening. Management involves addressing each anomaly individually. Propranolol is considered the first-line treatment for most cases of IH; however, it should be used with caution in PHACE syndrome, as hypotension may increase the risk of ischemic stroke.^23^ Lower doses of propranolol are recommended, and combination therapy with topical timolol is not advised.^24^

This case highlights the importance of emergency physicians considering dangerous diagnoses in patients with often benign and discounted presentations, such as IH.

## Funding and Support

By *JACEP Open* policy, all authors are required to disclose any and all commercial, financial, and other relationships in any way related to the subject of this article as per ICMJE conflict of interest guidelines (see www.icmje.org). The authors have stated that no such relationships exist.

## Conflict of Interest

All authors have affirmed they have no conflicts of interest to declare.
